# Perioperative extracorporeal membrane oxygenation in neonates with transposition of the great arteries: 15 years of experience

**DOI:** 10.1093/ejcts/ezae442

**Published:** 2025-01-22

**Authors:** Jesse A Weeda, Roel L F Van Der Palen, Heleen E Bunker-Wiersma, Lena Koers, Eelco Van Es, Mark G Hazekamp, Arjan B Te Pas, Peter Paul Roeleveld

**Affiliations:** Division of Pediatric Cardiology, Department of Pediatrics, Willem-Alexander Children’s Hospital, Leiden University Medical Centre, Leiden, the Netherlands; Division of Neonatology, Department of Pediatrics, Willem-Alexander Children’s Hospital, Leiden University Medical Centre, Leiden, the Netherlands; Division of Pediatric Cardiology, Department of Pediatrics, Willem-Alexander Children’s Hospital, Leiden University Medical Centre, Leiden, the Netherlands; Division of Pediatric Intensive Care, Department of Intensive Care, Leiden University Medical Centre, Leiden, the Netherlands; Division of Pediatric Intensive Care, Department of Intensive Care, Leiden University Medical Centre, Leiden, the Netherlands; Department of Cardiothoracic Surgery, Leiden University Medical Centre, Leiden, the Netherlands; Department of Cardiothoracic Surgery, Leiden University Medical Centre, Leiden, the Netherlands; Division of Neonatology, Department of Pediatrics, Willem-Alexander Children’s Hospital, Leiden University Medical Centre, Leiden, the Netherlands; Division of Pediatric Intensive Care, Department of Intensive Care, Leiden University Medical Centre, Leiden, the Netherlands

**Keywords:** Paediatric Intensive Care Units, Paediatric critical care, Transposition of the Great Vessels, Persistent pulmonary hypertension of the newborn, Extracorporeal Membrane Oxygenation

## Abstract

**OBJECTIVES:**

Extracorporeal membrane oxygenation (ECMO) can act as a bridge to recovery in both pre- and postoperative patients with transposition of the great arteries (TGA). However, literature on its use in these patients is scarce.

**METHODS:**

Retrospective single-centre cohort study encompassing all TGA patients who received ECMO between January 2009 and March 2024.

**RESULTS:**

Twenty-two neonates received ECMO during the study period, with an overall median age and weight at time of ECMO cannulation of 6.5 (1.8–10) days and 3.7 (3.3–4.0) kg, respectively. Twelve neonates received ECMO prior to the arterial switch operation because of severe persistent pulmonary hypertension (83%), respiratory failure due to severe pulmonary atelectasis (8%) or hypoxia after pulmonary arterial banding procedure (8%). Postoperative ECMO was used in 11 patients; of these, 1 (9%) had also received ECMO preoperatively. Postoperative indications for the remaining patients included failure to wean from cardiopulmonary bypass (50%), low cardiac output in Intensive Care Unit (20%), or after cardiopulmonary arrest (30%). Overall, median ECMO duration for all TGA patients was 75 (45–171) h, with a survival rate of 59% to hospital discharge. Among the preoperative ECMO patients, 5 patients (42%) died (4 preoperatively, 1 postoperatively performed while on ECMO). In the postoperative ECMO group, survival rate was 60%.

**CONCLUSIONS:**

In this single-centre retrospective study, TGA neonates received ECMO preoperatively primarily for severe pulmonary hypertension and postoperatively for failure to wean from cardiopulmonary bypass. This study showed a 58% and 60% survival to hospital discharge in ECMO patients supported preoperatively and those supported postoperatively, respectively.

## Graphical Abstract

**Figure:**
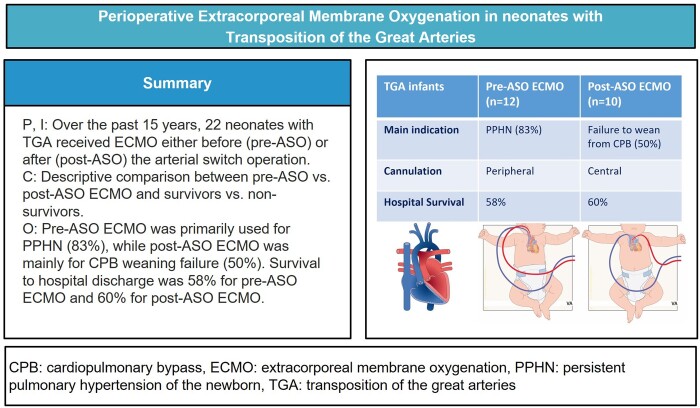

## INTRODUCTION

The use of extracorporeal membrane oxygenation (ECMO) can be an effective method of mechanical circulatory support for the management of severe pulmonary or cardiopulmonary failure [1]. Over the years, ECMO has evolved as an essential treatment available for children with congenital heart disease (CHD), such as transposition of the great arteries (TGA). TGA is characterized by concordant atrioventricular and discordant ventriculo-arterial connection, leading to a circulation in which the pulmonary and systemic circulation run in parallel rather than in series. The presence of an adequate communication between the 2 circulations [i.e. atrial septal defect or ventricular septal defect (VSD)] to allow mixing, along with a smooth foetal-to-neonatal transition involving a physiologic decrease of pulmonary vascular resistance, is crucial for adequate arterial saturation after birth [2]. However, neonates with TGA are at risk of developing pulmonary hypertension of the newborn (PPHN) after birth, especially those with an intact ventricular septum (TGA-IVS). In cases with severe PPHN or refractory cardiorespiratory failure, ECMO can offer a bridge to recovery while ensuring adequate systemic blood supply [3, 4].

In general, ECMO fulfils 3 primary purposes for children with cardiopulmonary failure: serving as a bridge to recovery, decision-making or therapeutic intervention [3, 4]. For TGA-patients, indications include preoperative stabilization (e.g. TGA with PPHN and subsequent hypoxia), failure to wean from cardiopulmonary bypass (CPB), low cardiac output in the Intensive Care Unit (ICU) postoperatively or after cardiopulmonary resuscitation (CPR) [5, 6]. As there are no randomized controlled trials in neonates with CHD and ECMO, there are no evidence-based guidelines for ECMO initiation, and our knowledge of ECMO support in these patients depends solely on retrospective single-centre and Extracorporeal Life Support Organization (ELSO) registry studies. Characterization, outcomes and complications of the use of ECMO in TGA patients have not been widely studied [7]. Therefore, the aim of this study is to describe our experience and outcomes in neonates with TGA supported perioperatively with ECMO over the past 15 years.

## MATERIALS AND METHODS

### Study population

This retrospective cohort study conducted at Leiden University Medical Centre includes all live-born neonates diagnosed with TGA who received ECMO support either before or after an arterial switch operation (ASO) between January 2009 and March 2024. Patients were either TGA-IVS, TGA with VSD or double-outlet right ventricle with subpulmonary VSD (Taussig-Bing anomaly). Coronary anatomy was scored as a usual coronary artery pattern (1LCx-2R and 1 l-2CxR) or alternate anatomies according to the Leiden Convention coronary coding system [8]. ECMO patients were divided into 2 subgroups: pre-arterial switch operation (pre-ASO) and post-arterial switch operation (post-ASO). Variables such as demographic data, gender, gestational age, morphological diagnosis, birth weight, surgical- and ECMO characteristics (procedural time, ECMO type and vascular access), haemodynamic course, extracorporeal CPR, ECMO-related complications and survival were obtained from the hospital records. Complications related to ECMO were assessed using complication codes established by the ELSO registry [4, 9]. The 1 patient who received both pre- and post-ASO ECMO support was assessed exclusively within pre-ASO group regarding the outcome parameters.

### Extracorporeal membrane oxygenation approach in transposition of the great arteries

The timing to initiate ECMO remains challenging, yet it should be initiated before signs of severe oxygen deficiency, organ damage or cardiac arrest in accordance with the guidelines outlined by the ELSO [10]. Veno-arterial ECMO (V-A ECMO) is the main support mode for ECMO in CHD and augments systemic cardiac output and respiratory gas exchange to facilitate adequate tissue oxygen delivery [3, 4]. V-A ECMO can be performed via peripheral cannulation (most commonly drainage via right internal jugular vein, return via right common carotid artery) or central cannulation (most commonly drainage via right atrium, return via ascending aorta) [10]. In our centre, peripheral V-A ECMO cannulation is typically performed in TGA-patients pre-ASO, whereas central cannulation is preferred for post-ASO patients. Desired ECMO flow is 100–150 ml/kg/min or 3 l/m^2^/min, titrated to achieve adequate oxygen delivery and lactate clearance. In case of significant cardiac failure with myocardial stunning and cardiac dilatation on ECMO, an extra cannula in the left atrium (i.e. left vent) or balloon atrial septostomy may be required to provide left ventricular decompression [11]. The approach in our institution is generally to wean infants from ECMO prior to the ASO. Weaning from ECMO is a complex process and requires careful assessment and timing, but can be initiated with signs of myocardial recovery and adequate resolution of systemic inflammatory response or pulmonary problems as per ELSO guidelines [12]. We generally start weaning ECMO-flow rates when lactate has normalized, pulse pressure is at least 15 mmHg, echocardiography suggests normalization of myocardial function and/or an RV-pressure is less than half systemic in children with PPHN, and pulmonary function seems adequate to allow for decannulation. If weaning is not successful despite several attempts, emergency ASO during ECMO support can be considered.

### Data management and analysis

Descriptive statistical analysis was performed using IBM SPSS (version 29; IBM SPSS Inc, Chicago, USA). All analyses were conducted using complete case analysis approach. Demographic and clinical data regarding procedure and complications were presented as frequency with percentages for categorical variables and mean with standard deviation or median with interquartile range for continuous data.

### Ethics

Approval was obtained from the appropriate ethics committee of the Leiden University Medical Centre (No. 2023-028). Committee waived the need for individual written informed consent.

## RESULTS

### Patient and extracorporeal membrane oxygenation characteristics

During the study period, 163 TGA patients were admitted to our centre, of whom 25 patients had PPHN (25/163; 15%) pre-ASO. All neonates with PPHN had TGA-IVS. Overall, 22 neonates received ECMO support (22/163; 13%), of whom (12/163; 7.4%) were assisted with ECMO before ASO (pre-ASO group). Four patients were deemed ineligible for ASO and died preoperatively without receiving ECMO (Supplementary Material, Table S1). Among them, a premature infant (gestational age 28 + 4 weeks, birth weight 1230 g) with a known prenatal TGA diagnosis died from multi-organ failure secondary to severe sepsis. The other 3 infants were diagnosed with TGA after birth and included the following: 3.5-month-old infant who died due to circulatory shock, compounded by progressive kidney injury and a middle cerebral artery infarction following delayed diagnosis; 2 infants who suffered severe neurological complications. One experienced a large subdural haematoma and extensive cerebral ischaemia, while the other had a severe genetic infantile motor neuron disease (AIFM1 mutation). One hundred fifty-five patients underwent an ASO, of whom 11 (7.1%) patients received ECMO support postoperatively (post-ASO group). There was 1 patient who received ECMO both before and after ASO and was included in the pre-ASO group. Baseline patient characteristics are outlined in Table 1. Overall, median age and weight at ECMO cannulation were 6.5 (interquartile range 1.8–10) days and 3.7 (interquartile range 3.2–4.0) kg, respectively. Morphological diagnosis consisted of TGA-IVS in 17 (77%), TGA with VSD in 1 (4.5%) and Taussig-Bing anomaly in 4 (18%) patients. All patients underwent V-A ECMO.

**Table 1:** Patient and ECMO characteristics

Patient characteristics	All patients (*n* = 22)	ECMO pre-ASO (*n* = 12)	ECMO post-ASO (*n* = 10)
Gestational age	39 + 3	39 + 4	39 + 2
Birthweight (kg)	3.6 (3.3–3.9)	3.7 (3.3–4.2)	3.4 (3.0–3.7)
Gender (male)	14 (64)	9 (75)	5 (50)
Morphological diagnosis			
TGA-IVS	17 (77)	10 (90)	6 (60)
TGA-VSD	1 (5)	0 (0)	1 (10)
TBA	4 (18)	1 (10)	3 (30)
Usual coronary anatomy (1LCx-2R, 1 l-2CxR)	14 (64)	10 (83)	4 (40)
Associated anomalies			
Aortic arch abnormality, aortic coarctation	3 (14)	1 (8)	2 (20)
Borderline LVOT	1 (5)	0 (0)	1 (10)
Balloon atrial septostomy	18 (82)	11 (92)	7 (70)
Prostaglandin E2 administration	21 (96)	12 (100)	9 (90)
ECMO characteristics			
Indication for ECMO			
PPHN	10	10	0
Respiratory failure atelectasis	1	1	0
Hypoxemia post-PAB	1	1	0
Failure to wean off CPB	5	0	5
Low cardiac output on ICU	2	0	2
Resuscitation in ICU	3	0	3
Age (days) at start ECMO	6.5 (1.8–10.0)	2.0 (1.0–2.8)	10 (8.5–16.0)
Weight at time of ECMO (kg)	3.7 (3.3–4.0)	3.8 (3.3–4.0)	3.5 (3.1–4.1)
Peak lactate prior to ECMO (<48 h) (mmol/l)	5.6 (4.0–7.2)	4.2 (3.4–5.2)	–
Peak lactate on ECMO (mmol/l)	8.1 (4.3–11.2)	5.3 (4.0–8.8)	9.8 (7.6–13.6)
Lowest pH prior to ECMO (<48 h)	7.15 (7.07–7.24)	7.17 (7.07–7.25)	–
Lowest pH on ECMO	7.16 (7.07–7.26)	7.23 (7.07–7.30)	7.13 (7.06–7.19)
ECMO duration (hours)	75 (41–171)	83 (51–171)	58 (30–191)
Left vent needed	4 (18)	0 (0)	4 (40)
Erythrocyte or platelet transfusion during ECMO	20 (91)	10 (83)	10 (100)

Data are in median (IQR) or *N* (%).

ASO: arterial switch operation; CPB: cardiopulmonary bypass time; CPR: cardiopulmonary resuscitation; DSC: delayed sternal closure; ECMO: extracorporeal membrane oxygenation; ICU: intensive care unit; IQR: interquartile range; LVOT: left ventricle outflow tract; NO: nitric oxide; PAB: pulmonary arterial banding; PPHN: persistent pulmonary hypertension of the newborn; TBA: Taussig-Bing anomaly; TGA: transposition of the great arteries; VSD: ventricular septal defect.

The main indication for TGA-patients with ECMO pre-ASO was PPHN (*n* = 10), with respiratory insufficiency due to severe pulmonary atelectasis (*n* = 1) or hypoxemia after pulmonary arterial banding (*n* = 1) as other indications. The incidence of ECMO in children with PPHN was 40% (10/25) with a mortality rate of 30% (3/10). All patients in the pre-ASO group were cannulated peripherally. Median weight, age and ECMO duration pre-ASO were 3.7 (3.3–4.2) kg, 2 (1.0–2.8) days and 83 (51–171) h, respectively (Table 1). Indications for ECMO after ASO were failure to wean off CPB (*n* = 5), low cardiac output syndrome in the ICU (*n* = 2) or after CPR in the ICU (*n* = 3). All patients were cannulated centrally, including the 1 patient who was also supported peripherally pre-ASO. Median weight, age and ECMO duration post-ASO were 3.5 (3.1–4.1) kg, 10 (8.5–16.0) days and 58 (30–191) h, respectively.

### Outcomes

**Figure 1:**
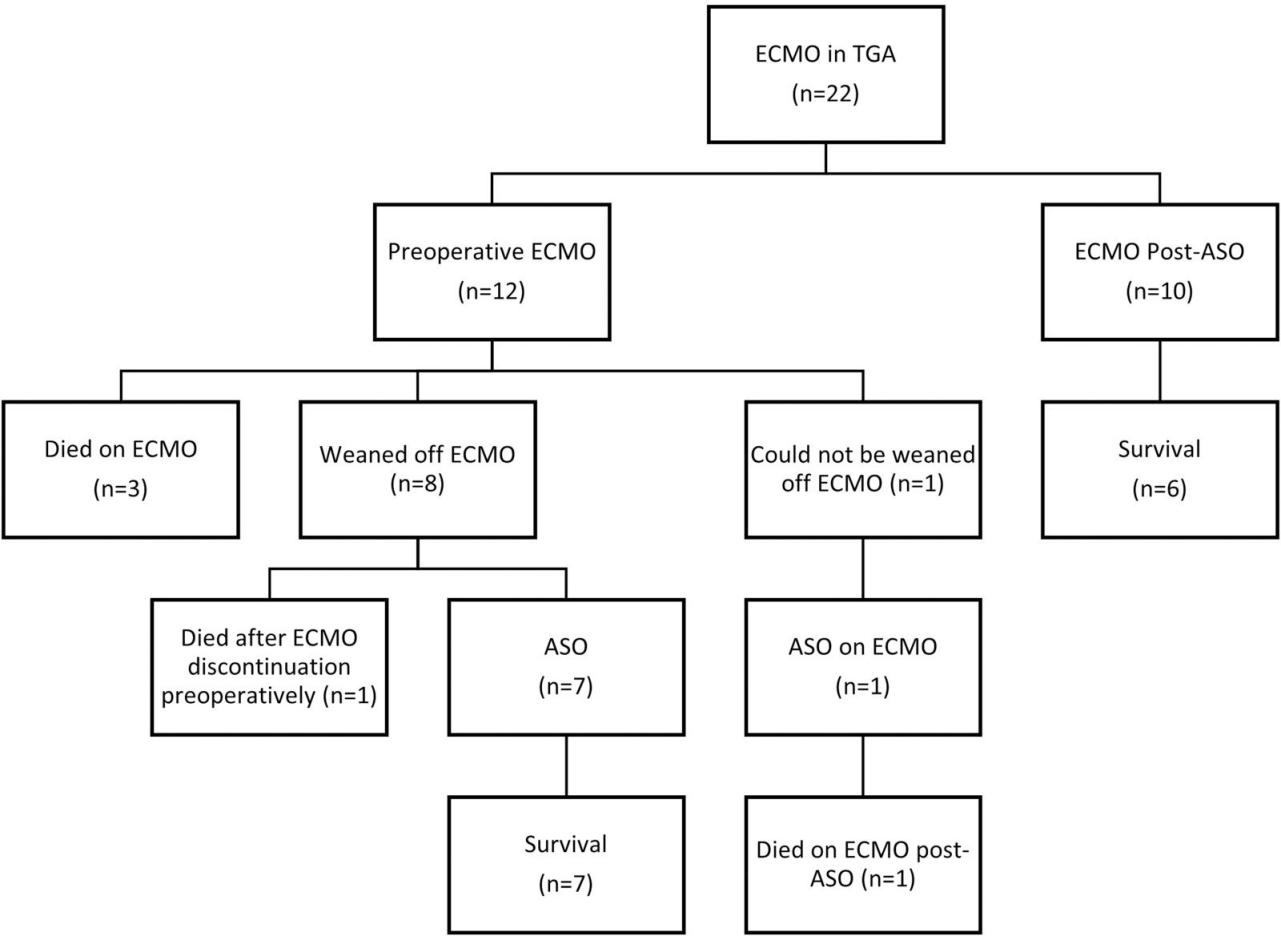
ECMO and survival. ASO: arterial switch operation; ECMO: extracorporeal membrane oxygenation; TGA: transposition of the great arteries.

Overall survival to hospital discharge in the ECMO group was 59% with a median length of ICU stay of 26 (17–48) days. Supplementary Material, Tables S1 and S2 provide demographic comparison between the entire TGA-cohort and the pre-and post-ASO ECMO groups. In the pre-ASO group, 8/12 (67%) were weaned of ECMO and 7/8 (88%) later underwent ASO (Fig. 1). Three patients died on ECMO and 1 patient could not be weaned off ECMO and underwent ASO on ECMO, continued ECMO support post-ASO and died thereafter (Fig. 1). In the pre-ASO ECMO group, survival rate was 7/12 (58%).

In the post-ASO ECMO group, survival rate was 6/10 (60%). In the post-ASO ECMO group, 3/10 (30%) patients underwent reoperation while on ECMO for various indications: coronary revision due to severely impaired left ventricular function in 1 patient, re-exploration due to bleeding of the neo-pulmonary artery wall with clot formation causing pressure on the right coronary artery in another patient and thrombectomy in the coronary anastomosis after (modified) Yacoub aortocoronary flap technique for single sinus coronary artery with intramural LAD (2 l*CxR), causing myocardial ischaemia with subsequent mitral valve repair in a 3rd patient. Only 1 survived to hospital discharge. No patients died post-ASO without having received ECMO (Supplementary Material, Table S2).

**Figure 2:**
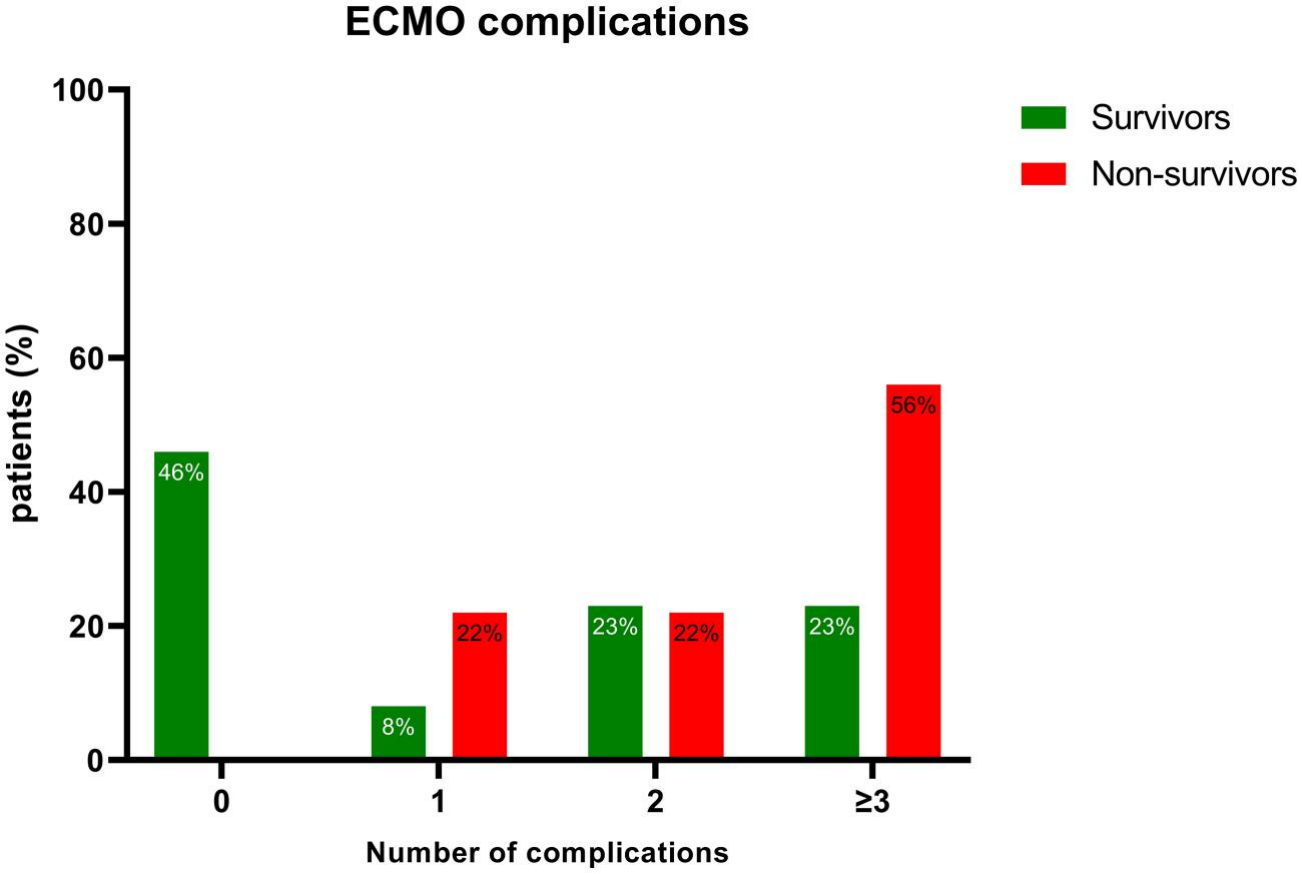
Number of ECMO complications across different domains between survivors and non-survivors. Total number of complications with ECMO across different domains (maximum score of 8) according to complication codes established by the ELSO registry [4, 12]. ECMO: extra corporeal membrane oxygenation.

Comparison of demographics between survivors and non-survivors are demonstrated in Table 2. In the survivor group, 46% of patients had no complications while on ECMO (Fig. 2); in the non-survivor group, 56% had complications in 3 or more organ domains. Complications across organ domains are outlined in Supplementary Material, Table S3. Non-survivors had more renal complications, as all neonates subjected to peritoneal dialysis or continuous veno-venous haemofiltration (4/22, 18%) were confined to the non-survivor group. There was a positive linear association between the duration of ECMO support and the number of ECMO complications across various complication categories (Supplementary Material, Fig. S1).

**Table 2:** Survivors versus non-survivors

Patient characteristics	Survivors (*n* = 13)	Non-survivors (*n* = 9)
Gestational age	39 + 3	39 + 4
Gender (male)	8 (62)	6 (67)
Birthweight (kg)	3.4 (3.1–3.8)	3.7 (3.4–4.2)
Morphological diagnosis		
TGA-IVS	10	7
TGA-VSD	1	0
TBA	2	2
Usual coronary anatomy (1LCx-2R, 1 l-2CxR)	9 (69)	5 (55)
Associated Anomalies	1	3
Aortic arch abnormality, aortic coarctation	0	3
Borderline LVOT	0	1
Balloon atrial septostomy	10 (77)	8 (89)
Prostaglandin E2 administration	12 (92)	9 (100)
Pulmonary hypertension (need for NO)		
Pre-ASO	7 (54)	5 (56)
Post-ASO	1 (11)	3 (23)
CPR pre-ECMO	2 (15)	2 (22)
Total intubation time (days)	25 (13–35)	19 (6–44)
ICU admission		
Post-ASO (days)	14 (12–39)	16 (5–23)
Total ICU admission (days)^a^	29 (20–65)	19 (3–44)
ECMO characteristics		
Age (days) at start ECMO	3 (2–12)	7 (2–9)
Weight at time of ECMO (kg)	3.5 (3.0–3.9)	4.0 (3.5–4.2)
Peak lactate prior to ECMO (<48 h) (mmol/l)	4.6 (4.1–6.4)	7.0 (3.4–11.9)
Peak lactate on ECMO (mmol/l)	4.5 (3.9–9.6)	9.0 (7.8–13.4)
Lowest pH prior to ECMO	7.19 (7.10–7.26)	7.08 (7.01–7.11)
Lowest pH on ECMO (<48 h)	7.22 (7.08–7.29)	7.12 (7.06–7.22)
ECMO duration (hours)	78 (50–102)	49 (25–447)
Left vent needed	1 (8)	3 (33)
Erythrocyte or platelet transfusion during ECMO	13 (100)	7 (78)
ASO characteristics		
CPB (min)	188 (140–244)	229 (189–372)
Aortic cross-clamping time (min)	95 (89–139)	125 (92–207)

Data are in median (IQR) or *N* (%).

a Total ICU admission (days): total duration of ICU admission including pre- and post-ASO time.

ASO: arterial switch operation; CPB: cardiopulmonary bypass time; CPR: cardiopulmonary resuscitation; ECMO: extracorporeal membrane oxygenation; ICU: intensive care unit; IQR: interquartile range; LVOT: left ventricle outflow tract; NO: nitric oxide; PAB: pulmonary arterial banding; PPHN: persistent pulmonary hypertension of the newborn; TBA: Taussig-Bing anomaly; TGA: transposition of the great arteries; VSD: ventricular septal defect.

## DISCUSSION

Over the years, ECMO has evolved as an essential support modality for neonates with TGA experiencing severe cardiorespiratory failure. ECMO can act as a bridge to recovery in both pre- and post-ASO TGA-management. This single-centre retrospective study showed a 58–60% hospital survival in neonates supported with ECMO before and/or after ASO, respectively.

### Pre-arterial switch operation extracorporeal membrane oxygenation support

PPHN was the most frequently observed indication for pre-ASO ECMO support. Previous reports have associated TGA with PPHN, with incidence rates up to 21.5% [13, 14]. Similarly, the incidence of PPHN in our entire cohort of TGA patients was 15% (25/163), of which 10 (40%) received ECMO. As in our cohort, PPHN was predominantly observed in the subtype of TGA patients with intact ventricular septum [13, 14]. Exact causes for severe PPHN in these patients have not been elucidated so far. In literature, suggestions have been made indicating a complex multifactorial aetiology. Contributing factors might include: (i) hypoxemia and acidosis during the intrauterine or the early postnatal period; (ii) restrictive oval fossa or foetal ductal constriction resulting from increased oxygen content in the pulmonary artery in foetuses with TGA; (iii) increased pulmonary artery wall thickness and muscularity with intimal proliferation; (iv) mediating pathways such as endothelin-1, prostacyclin-cGMP, nitric oxide-cAMP and vascular endothelial growth factor; and/or (v) clinically unrecognized pulmonary microthrombi [13, 15–17].

Conventional treatment of PPHN includes adequate sedation and analgesia, mechanical ventilation, inhaled nitric oxide and haemodynamic support with fluids and inotropes including milrinone and/or balloon atrial septostomy. Our approach has not changed significantly over the course of the study period. In TGA-patients with PPHN, resistant to conventional treatment, ECMO can be used as cardiopulmonary support. Nevertheless, PPHN in neonates with TGA has been associated with significant morbidity and preoperative mortality rates up to 29% in series with and without ECMO use [13]. In a previous report of PPHN in TGA-patients by Sallaam *et al*. [14], 45% underwent ECMO support pre-ASO, with a mortality rate of 44% within this ECMO subgroup. This is similar to our cohort in which 40% of neonates with PPHN received ECMO with a mortality rate of 40%.

Pre-ASO ECMO in infants with PPHN is most often used as a bridge to ASO, yet the optimal timing of ASO in relation to ECMO remains unclear.

During ASO, CPB and stress of surgery and anaesthesia may cause a further increase of pulmonary vascular resistance, as well as a decrease of myocardial function, potentially leading to insufficient cardiopulmonary function postoperatively [14]. Therefore, the approach in our centre is to wean patients of ECMO prior to ASO as also advocated by *Jaillard* et al. [5], allowing for gradual reconditioning of the myocardium to prevent postoperative deterioration related to recurrent PPHN. However, other studies suggest to perform ‘rescue ASO’ while on ECMO to maintain end-organ function [18]. In our cohort, all but 1 patient was successfully weaned off ECMO before surgery. All our pre-ASO ECMO patients were supported with V-A ECMO and cannulated peripherally. V-venous ECMO has also successfully been described [19].

### Post-arterial switch operation extracorporeal membrane oxygenation support

The ELSO guidelines emphasize the importance of timely ECMO indication postoperatively [3]. According to data from the Congenital Heart Surgery Database of the Society of Thoracic Surgery, the usage of ECMO support in neonates following ASO varies between 3.7% and 7.5%, depending on whether VSD repair was conducted (7.5%) or not (3.7%) [20]. In our cohort, 7.1% of all ASO patients received post-ASO ECMO, most of them (50%) because of failure to wean from CPB. All were cannulated centrally. Failure to wean from CPB may be influenced by several factors such as residual PPHN, inadequate myocardial function, or technical issues associated to the surgical procedure [21]. Causes for low cardiac output post-CPB are myocardial ischaemia (typically caused by technical problems of coronary artery transfer) or the inability of the left ventricle to adapt to systemic pressures (often referred to as a detrained left ventricle) post-ASO. Determining the exact timing for ECMO initiation after surgical repair of CHD in general remains challenging, with conflicting findings on hospital survival [22]. While some studies report improved survival rates when ECMO is initiated in the operating room, others indicate no disparity in survival based on the timing of ECMO initiation postoperatively [21].

Timely diagnosis and intervention of unexpected residual lesions are associated with improved outcomes in post-cardiotomy ECMO patients in general [23, 24]. Yang *et al*. [7] identified coronary abnormalities, such as intramural, solitary coronary artery and mono-coronary ostium, as influential factors on the outcomes of TGA-patients undergoing ECMO in the intraoperative and postoperative subgroup. In our series, 60% of the patients receiving post-ASO ECMO support exhibited a variant in coronary artery anatomy. This finding potentially reinforces the hypothesis that non-typical coronary anatomy contributes to increased surgical complexity.

### Outcomes

In our cohort, survival to hospital discharge was 59% and consistent in both pre-ASO (58%) and post-ASO (60%) ECMO patients. The average survival for neonates with all types of CHD supported with ECMO is 40–51% as reported by the ELSO registry database [3].

Various predictors for mortality on ECMO after surgery for CHD have previously been identified in literature and include young age, low weight, high inotrope score, high lactate, acidosis, duration of ventilation, presence of fluid overload, renal failure, dialysis, cardiac catheterization, CPR requirement and ECMO duration. In this cohort, we observed that non-survivors had more renal complications, compared to survivors. The association between ECMO, renal complications and mortality has been previously recognized; therefore, renal failure could serve as an indicator of poor prognosis while on ECMO support [25].

The timing and duration of ECMO play an important role, as prolonged ECMO duration has been associated with increased complications, such as renal failure, bleeding, thrombosis and infection, adversely affecting survival [26]. Based on the ELSO registry study, the overall survival rate of paediatric cardiac ECMO runs between 2000 and 2011 was 45%, but survival decreased to 23–25% for ECMO durations between 14 and 28 days, and further dropped to 13% for ECMO runs lasting longer than 28 days [27]. Consistent with these findings, we also observed a higher complication rate and involvement of multiple organs systems with prolonged ECMO duration. However, due to the cohort’s limited sample size and statistical power, we were unable to identify a definitive relationship between ECMO duration and outcomes.

### Limitations

This study is subject to the limitations inherent to a retrospective single centre design, despite having no missing data. The small sample size limits statistical power leading us to provide only descriptive statistics. Additionally, the study period of more than a decade, along with technological advances in ECMO equipment and our increased experience with postcardiotomy ECMO, could have influenced ECMO-initiation thresholds and outcomes. In the first 5 years of the study period, we only supported 3 TGA patients with ECMO possibly indicative of our increased threshold at the start of our ECMO program, but in the last 10 years, we experienced a relatively steady number of annual ECMO runs for patients with TGA, suggesting a well-balanced approach to these specific patients. Unfortunately, the cohort is too small to analyse this effect of time on outcomes. However, for postcardiotomy, ECMO in general outcomes have also been relatively stable over the last 15 years according to the ELSO registry [4].

## CONCLUSION

This single-centre retrospective study over a 15-year period of ECMO in neonates with TGA, showed a 58 and 60% survival to hospital discharge in patients supported pre-ASO and those supported post-ASO, respectively. Most pre-ASO patients received ECMO due to PPHN and post-ASO patients due to failure to wean off CPB.

## Supplementary Material

ezae442_Supplementary_Data

## Data Availability

Databases will be made available from the corresponding author upon reasonable request.

## ABBREVIATIONS

ASO: Arterial switch operation
CHD: Congenital heart disease
CPB: Cardiopulmonary bypass
CPR: Cardiopulmonary resuscitation
ECMO: Extracorporeal membrane oxygenation
ELSO: Extracorporeal Life Support Organization
ICU: Intensive care unit
PPHN: Persistent pulmonary hypertension of the newborn
pre-ASO: Pre-arterial switch operation
TGA: Transposition of the great arteries
TGA-IVS: TGA with intact ventricular septum
V-A ECMO: Veno-arterial ECMO

## References

[1] Erdil T , Lemme F, Konetzka A et al Extracorporeal membrane oxygenation support in pediatrics. Ann Cardiothorac Surg 2019;8:109–15. 30854319 10.21037/acs.2018.09.08 PMC6379197

[2] Szymanski MW , Moore SM, Kritzmire SM, Goyal A, Transposition of the great arteries. In: StatPearls. Treasure Island, FL: StatPearls Publishing, 2023. 30860704

[3] Roeleveld PP , Mendonca M. Neonatal cardiac ECMO in 2019 and beyond. Front Pediatr 2019;7:327. 31497583 10.3389/fped.2019.00327 PMC6712998

[4] Organization ELSO: Registry Report International Summary. Extracorporeal Life Support Organization. 2023. https://www.elso.org/Registry/Statistics/InternationalSummary (January 2024, date last accessed).

[5] Jaillard S , Belli E, Rakza T et al Preoperative ECMO in transposition of the great arteries with persistent pulmonary hypertension. Ann Thorac Surg 2005;79:2155–8. 15919337 10.1016/j.athoracsur.2003.12.037

[6] Salvin JW , Laussen PC, Thiagarajan RR. Extracorporeal membrane oxygenation for postcardiotomy mechanical cardiovascular support in children with congenital heart disease. Paediatr Anaesth 2008;18:1157–62. 19076568 10.1111/j.1460-9592.2008.02795.x

[7] Yang L , Ye L, Yu J et al Lessons learned from ECMO support in pediatric patients with D-transposition of the great arteries: preoperative, intraoperative and postoperative. World J Pediatr Surg 2021;4:e000273. 36475240 10.1136/wjps-2021-000273 PMC9716810

[8] Gittenberger-de Groot AC , Koenraadt WMC, Bartelings MM et al Coding of coronary arterial origin and branching in congenital heart disease: the modified Leiden Convention. J Thorac Cardiovasc Surg 2018;156:2260–9. 30243713 10.1016/j.jtcvs.2018.08.009

[9] Thiagarajan RR , Laussen PC, Rycus PT, Bartlett RH, Bratton SL. Extracorporeal membrane oxygenation to aid cardiopulmonary resuscitation in infants and children. Circulation 2007;116:1693–700. 17893278 10.1161/CIRCULATIONAHA.106.680678

[10] Brown G , Moynihan KM, Deatrick KB et al Extracorporeal Life Support Organization (ELSO): guidelines for pediatric cardiac failure. [published correction appears in ASAIO J. 2022 1;68:e129]. ASAIO J 2021;67:463–75. 33788796 10.1097/MAT.0000000000001431

[11] Xie A , Forrest P, Loforte A. Left ventricular decompression in veno-arterial extracorporeal membrane oxygenation. Ann Cardiothorac Surg 2019;8:9–18. 30854308 10.21037/acs.2018.11.07 PMC6379183

[12] Aissaoui N , Luyt CE, Leprince P et al Predictors of successful extracorporeal membrane oxygenation (ECMO) weaning after assistance for refractory cardiogenic shock. Intensive Care Med 2011;37:1738–45. 21965097 10.1007/s00134-011-2358-2

[13] Roofthooft MT , Bergman KA, Waterbolk TW, Ebels T, Bartelds B, Berger RM. Persistent pulmonary hypertension of the newborn with transposition of the great arteries. Ann Thorac Surg 2007;83:1446–50. 17383355 10.1016/j.athoracsur.2006.11.001

[14] Sallaam S , Natarajan G, Aggarwal S. Persistent pulmonary hypertension of the newborn with D-transposition of the great arteries: management and prognosis. Congenit Heart Dis 2016;11:239–44. 26554402 10.1111/chd.12304

[15] Kumar A , Taylor GP, Sandor GG, Patterson MW. Pulmonary vascular disease in neonates with transposition of the great arteries and intact ventricular septum. Br Heart J 1993;69:442–5. 8518068 10.1136/hrt.69.5.442 PMC1025109

[16] Newfeld EA , Paul MM, Muster AJ, Idriss FS. Pulmonary vascular disease in complete transposition of the great arteries: a study of 200 patients. Am J Cardiol 1974;34:75–82. 4835757 10.1016/0002-9149(74)90096-4

[17] Geiger R , Berger RM, Hess J, Bogers AJ, Sharma HS, Mooi WJ. Enhanced expression of vascular endothelial growth factor in pulmonary plexogenic arteriopathy due to congenital heart disease. J Pathol 2000;191:202–7. 10861582 10.1002/(SICI)1096-9896(200006)191:2<202::AID-PATH608>3.0.CO;2-D

[18] Luciani GB , Chang AC, Starnes VA. Surgical repair of transposition of the great arteries in neonates with persistent pulmonary hypertension. Ann Thorac Surg 1996;61:800–5. 8619696 10.1016/0003-4975(95)01089-0

[19] Yam N , Chen RH, Rocha BA, Lun KS, Yung TC, Au TW. Preoperative venovenous extracorporal membrane oxygenation for transposition of great arteries with severe pulmonary hypertension in a newborn. Ann Thorac Surg 2020;109:e329–e330. 31586616 10.1016/j.athoracsur.2019.08.077

[20] Mascio CE , Austin EH 3rd, Jacobs JP et al Perioperative mechanical circulatory support in children: an analysis of the Society of Thoracic Surgeons Congenital Heart Surgery Database. J Thorac Cardiovasc Surg 2014;147:658–65. 24246548 10.1016/j.jtcvs.2013.09.075 PMC3926808

[21] Kolovos NS , Bratton SL, Moler FW et al Outcome of pediatric patients treated with extracorporeal life support after cardiac surgery. Ann Thorac Surg 2003;76:1435–42. 14602263 10.1016/s0003-4975(03)00898-1

[22] Gupta P , Robertson MJ, Rettiganti M et al Impact of timing of ECMO initiation on outcomes after pediatric heart surgery: a multi-institutional analysis. Pediatr Cardiol 2016;37:971–8. 27037549 10.1007/s00246-016-1379-6

[23] Agarwal HS , Hardison DC, Saville BR et al Residual lesions in postoperative pediatric cardiac surgery patients receiving extracorporeal membrane oxygenation support. J Thorac Cardiovasc Surg 2014;147:434–41. 23597724 10.1016/j.jtcvs.2013.03.021

[24] Soynov IA , Kornilov IA, Kulyabin YY et al Residual lesion diagnostics in pediatric postcardiotomy extracorporeal membrane oxygenation and its outcomes. World J Pediatr Congenit Heart Surg 2021;12:605–13. 34597209 10.1177/21501351211026594

[25] Guerguerian AM , Sano M, Todd M, Honjo O, Alexander P, Raman L. Pediatric extracorporeal cardiopulmonary resuscitation ELSO guidelines. [published correction appears in ASAIO J. 2022;68:e130]. ASAIO J 2021;67:229–37. 33627593 10.1097/MAT.0000000000001345

[26] Alsoufi B , Slesnick T, McCracken C et al Current outcomes of the Norwood operation in patients with single-ventricle malformations other than hypoplastic left heart syndrome. World J Pediatr Congenit Heart Surg 2015;6:46–52. 25548343 10.1177/2150135114558069

[27] Merrill ED , Schoeneberg L, Sandesara P et al Outcomes after prolonged extracorporeal membrane oxygenation support in children with cardiac disease—Extracorporeal Life Support Organization registry study. J Thorac Cardiovasc Surg 2014;148:582–8. 24189317 10.1016/j.jtcvs.2013.09.038

