# Supplementary figures and images for: Perioperative extracorporeal membrane oxygenation in neonates with transposition of the great arteries: 15 years of experience
Source: Eur J Cardiothorac Surg. 2025 Jan 22;67(2):ezae442. doi: 10.1093/ejcts/ezae442 (PMC11814491; doi:10.1093/ejcts/ezae442)

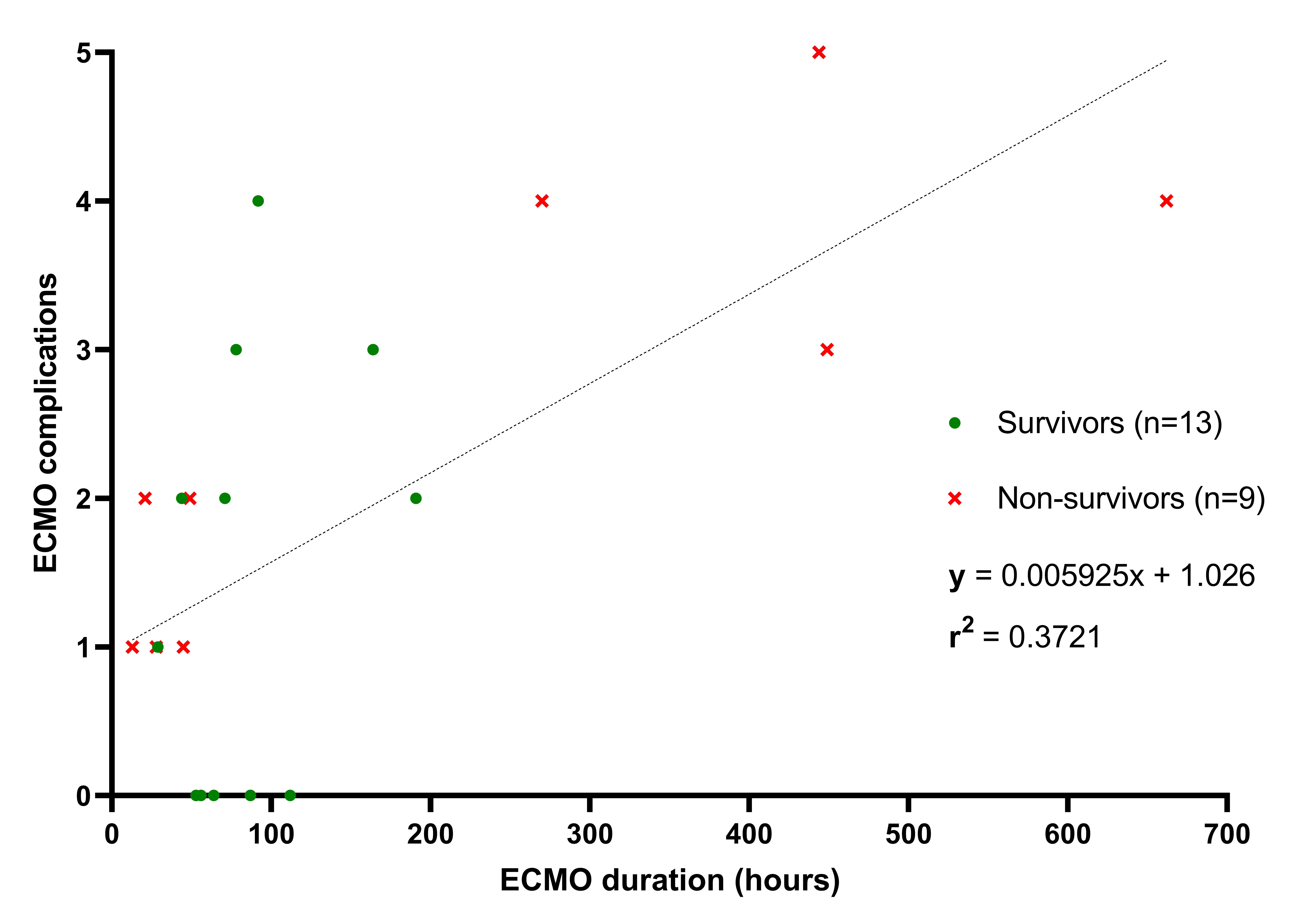

Supplement: ezae442_Supplementary_Data [file ezae442_supplementary_data.jpeg]
